# Effects on Metallization of n^+^-Poly-Si Layer for N-Type Tunnel Oxide Passivated Contact Solar Cells

**DOI:** 10.3390/ma17112747

**Published:** 2024-06-05

**Authors:** Qinqin Wang, Beibei Gao, Wangping Wu, Kaiyuan Guo, Wei Huang, Jianning Ding

**Affiliations:** 1 Institute of Technology for Carbon Neutralization, Yangzhou University, Yangzhou 225009, China; wqqwh517@126.com (B.G.); mz120220597@stu.yzu.edu.cn (K.G.); mx120220364@stu.yzu.edu.cn (W.H.); 2 Jinko Solar Co., Ltd., Haining 314400, China; 3 Electrochemistry and Corrosion Laboratory, School of Mechanical Engineering, Changzhou University, Changzhou 213164, China; wwp3.14@163.com

**Keywords:** n^+^-poly-Si layer, n-TOPCon solar cell, metallization, parasitic absorption, Ag pastel

## Abstract

Thin polysilicon (poly-Si)-based passivating contacts can reduce parasitic absorption and the cost of n-TOPCon solar cells. Herein, n^+^-poly-Si layers with thicknesses of 30~100 nm were fabricated by low-pressure chemical vapor deposition (LPCVD) to create passivating contacts. We investigated the effect of n^+^-poly-Si layer thickness on the microstructure of the metallization contact formation, passivation, and electronic performance of n-TOPCon solar cells. The thickness of the poly-Si layer significantly affected the passivation of metallization-induced recombination under the metal contact (*J*_0,*metal*_) and the contact resistivity (*ρ_c_*) of the cells. However, it had a minimal impact on the short-circuit current density (*J_sc_*), which was primarily associated with corroded silver (Ag) at depths of the n^+^-poly-Si layer exceeding 40 nm. We introduced a thin n^+^-poly-Si layer with a thickness of 70 nm and a surface concentration of 5 × 10^20^ atoms/cm^3^. This layer can meet the requirements for low *J*_0,*metal*_ and *ρ_c_* values, leading to an increase in conversion efficiency of 25.65%. This optimized process of depositing a phosphorus-doped poly-Si layer can be commercially applied in photovoltaics to reduce processing times and lower costs.

## 1. Introduction

A tunnel oxide passivated contact (TOPCon) solar cell with an ultrathin silicon oxide (SiO*_x_*) film and a phosphorus-doped polysilicon (n^+^-poly-Si) layer has the potential for a high theoretical efficiency limit of 28.7% [1]. The market share of TOPCon cells is significant according to the ITRPV [2]. An n-TOPCon cell (size: 330.15 cm^2^) at JinkoSolar has achieved the highest conversion efficiency recorded thus far, 26.89% [3]. The n^+^-poly-Si/SiO*_x_* layer with high carrier selectivity exhibits superior passivation quality at the metal/silicon contact, distinguishing it from Passivated Emitter and Rear Cell (PERC) solar cells [4,5]. The ultrathin SiO*_x_* layer has a large tunneling barrier for holes [6]. The doped poly-Si layer separates the metal/silicon contact from minority charge carriers, resulting in low recombination and band bending. The doped poly-Si is typically crystallized from an amorphous silicon layer (a-Si) after a high-temperature heat treatment process. Normally, the desired a-Si layer can be prepared using methods such as plasma-enhanced chemical vapor deposition (PECVD) [7,8], low-pressure CVD (LPCVD) [9,10], atmospheric pressure CVD [11], hot wire CVD [12], sputtering [13,14], and electron beam evaporation [15]. Published studies focused on poly-Si passivating contacts fabricated using the standard industrial approach based on LPCVD and ex situ phosphorus (P) doping. This process has high throughput and is simple [4,16,17,18,19,20]. Studies in the literature investigated the parameters of passivating contacts, including a tunneling SiO*_x_* film [21], the structural properties and thickness of the poly-Si film [22,23,24], and the metallization of the passivating contact [16]. These studies have provided valuable insights into the impacts of LPCVD process parameters on the structure and electrical properties of poly-Si films [17,25,26,27,28,29]. 

The doped poly-Si layer could be helpful in achieving good passivation performance and low contact resistivity by controlling interfacial defects in the SiO*_x_* and the doping concentration of the poly-Si layer [30,31]. After maximizing contact passivation using a SiO*_x_*/poly-Si layer, the lowest dark saturation current densities (*J*_0_) of emitters in cells with passivation layers comprising an n^+^-poly-Si/SiO*_x_* contact and a boron-doped poly-Si/SiO*_x_* contact were 0.66 fA/cm^2^ and 4.4 fA/cm^2^, respectively [32]. At present, researchers and engineers are making further efforts to improve cell efficiency. One important method is minimizing the parasitic absorption losses of the poly-Si layer for solar cells. However, a poly-Si layer with a thickness of 100 nm is necessary under current metallization conditions [4,33]. At the same time, various studies have been conducted to prevent metal finger penetration by using a barrier interlayer, such as titanium nitride (TiN) [34,35] or SiO*_x_* [36,37], created through a low-temperature method like Laser-Enhanced Contact Optimization (LECO) [38]. Although there are many positive outcomes regarding the formation of planar (n) poly-Si passivating contacts, one of the challenging and critical tasks associated with poly-Si layers is determining how to balance parasitic absorption and contact in poly-Si layers [24,39,40,41]. 

In this work, n-TOPCon cells containing P-doped poly-Si with passivating contacts were fabricated. The thickness of the poly-Si layer was changed using the LPCVD method, and the influence of the poly-Si layer thickness on *J*_0_, metallization recombination (*J*_0,*metal*_), and contact resistivity (*ρ_c_*) were studied; a microstructure analysis of the contact formation was conducted; and further, the *I*–*V* parameters of the n-TOPCon solar cells were investigated, including their efficiency (*E_ff_*), open-circuit voltage (*V_oc_*), fill factor (FF), series resistance (*R_ser_*), and short-circuit current density (*J_sc_*). 

## 2. Experimental 

### 2.1. Fabrication

**Figure 1:**
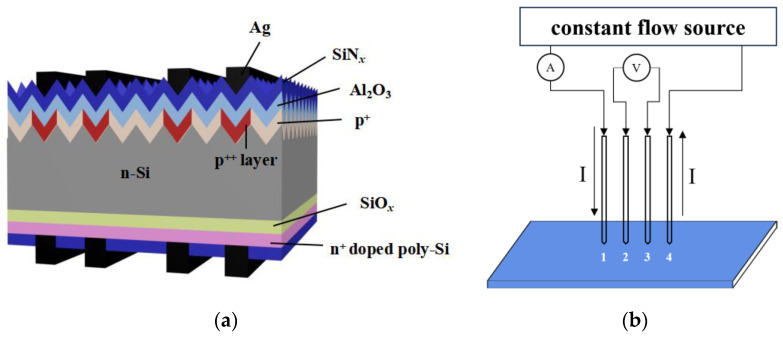
(**a**) Structure of n-TOPCon solar cell and (**b**) schematic diagram of four-probe sheet resistance tester.

n-TOPCon cells were obtained from commercially available n-type Czochralski silicon (Cz-Si) wafers. Texture was generated in an alkaline (KOH)/textured additive solution. The textured wafers were placed into a quartz tube furnace containing BCl_3_ gas using a LYDOP™ system. Subsequently, the B-doping side was treated with a Hymson laser with a beam width of 90–100 μm to obtain front B-selective emitters. After the laser treatment, the rear side was cleansed with a KOH/polished additive solution. One 1.6 ± 0.2 nm thick layer of SiO*_x_* and an intrinsic a-Si 30–100 nm thick layer on the rear side were prepared by LPCVD. Subsequently, the a-Si layer was crystallized to form poly-Si in a high-temperature tube furnace at 850 °C for 20–30 min using a gas mixture of POCl_3_, O_2_, and N_2_ which could be simultaneously doped with P. Phosphorus silicate glass (PSG) was removed using a 5% HF solution for 5 min, and the P-doping concentration profiles of the SiO*_x_*/P-doped poly-Si films were determined by electrochemical capacitance–voltage (ECV) profiling. Wafers with different P-doped poly-Si film thicknesses using two-sided passivation of the SiN*_x_* layer were used as *J*_0_ samples. Similar samples with polished surfaces were screen-printed with Ag paste lines on one side only and nine different pitches were used as *J*_0,*metal*_ samples; these which were placed with the fingers facing up and then sintered in a sintering furnace at a peak temperature of 730 °C [42,43,44]. P-doped poly-Si contacts were fabricated with four different thicknesses (see Table 1). Figure 1 displays the structure of an n-TOPCon solar cell.

After cleaning the wafers and removing the BSG and PSG, a clean P-doped poly-Si/SiO*_x_* layer was obtained on one side only by etching the poly-Si wraparound side in a mixed solution of KOH and polishing additives. The front and rear of the cells were passivated with 4 nm thick Al_2_O_3_ through ALD/78 nm thick SiN*_x_* and 75 nm thick SiN*_x_* through PECVD, respectively. The fingers and busbars of the wafers were metallized using a commercial Ag paste, using an H-patterned grid which was screen-printed on both sides with a 16-busbar configuration. Finally, the cells with Ag fingers were heat-treated at a peak temperature of 730 °C.

### 2.2. Characterization

The current–voltage (*I*–*V*) parameters of the in-house standard cells were measured using a Wavelabs tester. The *J*_0_ and *J*_0,*metal*_ values of the samples were measured using a WCT-120 Sinton and extracted at an excess carrier density of 3 × 10^15^ cm^−3^ (Boulder, CO, USA) [45]. The SiO*_x_*/P-doped poly-Si profiles of the monitor wafers were measured by an ECV device (WEP CVP21), using a 0.1 M NH_4_F solution as an etchant. We used a four-probe test rig to determine the sheet resistance (Figure 1b). The *ρ_c_* values were measured using the transfer-length method (TLM; GP-4 TEST) from the H-patterns printed on one side of the *J*_0_ samples. The microstructures and chemical composition of the contact interfaces were observed by energy-filtered scanning electron microscopy (FESEM-EDS, Regulus8230, HITCHI, Tokyo, Japan) and transmission electron microscopy (Thermo Scientific Talos™ STEM, 200 kV, FEI Talos F200X, Waltham, MA, USA). The optical reflectance values and losses of the cells were analyzed using a PVE300-IVT (Bentham Instruments, Reading, UK) instrument and the Current Loss Analysis Calculator V1.4 (Series, from the Solar Energy Research Institute of Singapore, based on the Yablonovitch limit of 46.43 mA/cm^2^ [46]).

## 3. Results and Discussion

### 3.1. Microstructure and Performance

**Figure 2:**
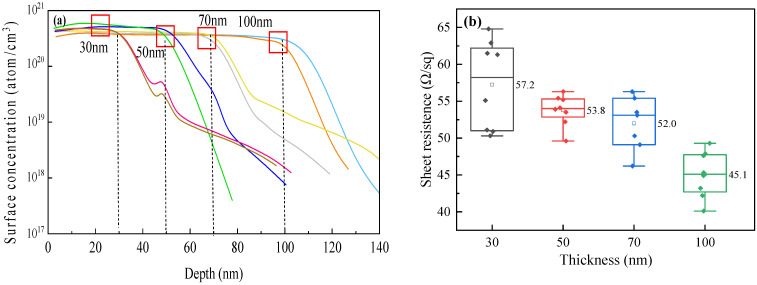
ECV profiles (**a**) and sheet resistances (**b**) of cells with different n^+^-poly-Si/SiO*_x_* layer thicknesses. (The solid pattern represents the test data, and the hollow pattern represents the average value).

The n^+^-poly-Si layers of different thicknesses influenced the n^+^-poly-Si/SiO*_x_* profiles, sheet resistance (*R_□_*), *J*_0_, *ρ_c_*, and *J*_0,*metal*_ of the n-TOPCon cells, and the results are shown in Table 1. Figure 2 displays the ECV profiles and sheet resistances for different thicknesses of n^+^-poly-Si/SiO*_x_* layers. In Figure 2a, n^+^-poly-Si layers with different thicknesses have almost the same surface concentration of >5 × 10^20^ atoms/cm^3^ and nearly the same “knee-shaped” tail ECV profile; these were doped at the same high temperature. On an n-type silicon wafer, when the thickness of the n^+^-poly-Si layer was reduced from 100 to 30 nm, the sheet resistance (*R_□_*) of the cell increased from 45 to 57 Ω/sq. This increase occurred because the thickness of the poly-Si layer determined the total doping amount (Figure 2b). Although the surface doping concentration is the same, ECV curves of poly-Si layers with different thickness are integrated into the total doping amount. It can be found that as the thickness of the poly-Si layer increases, the total doping amount also increases, leading to a decrease in sheet resistance.

**Figure 3:**
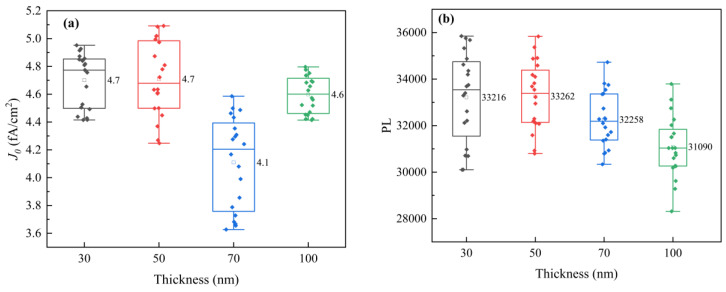
*J*_0_ (**a**) and PL (**b**) plots of cells. (The solid pattern represents the test data, and the hollow pattern represents the average value).

The *J*_0_ values of n^+^-poly-Si/SiO*_x_* layers with different poly-Si thicknesses exhibit a slight change of approximately 0.5 fA/cm^2^ when the thickness increases from 30 to 100 nm. The penetration depth is determined from the inflection point of the SiO*_x_* layer to a doping concentration of N = 1 × 10^19^ atoms/cm^3^ (Figure 3). It can be seen from Figure 3a that as the poly-Si thickness increases, the penetration depth of p-doping increases, and the inflection point concentration decreases. When the thickness of the poly-Si layer is 30 nm, the inflection point concentration is higher, resulting in a large Auger recombination so the *J*_0_ value becomes large. When the thickness of the poly-Si layer is 100 nm, the inflection point concentration is low, the passivation effect is poor, and *J*_0_ increases. In order to study the passivation properties of different poly-Si thicknesses, PL tests were performed. The results showed that PL intensity decreased slightly with an increase in thickness. The PL values also showed a minor change of approximately 1000 a.u. This indicates that compared to a 100 nm thick layer of n^+^-poly-Si/SiO*_x_*, a 30 nm thick layer can also provide effective field-effect passivation. This is attributed to the good passivation of the SiO*_x_* layer and a 15~20 nm penetration depth of P doped into the Si substrate.

**Figure 4:**
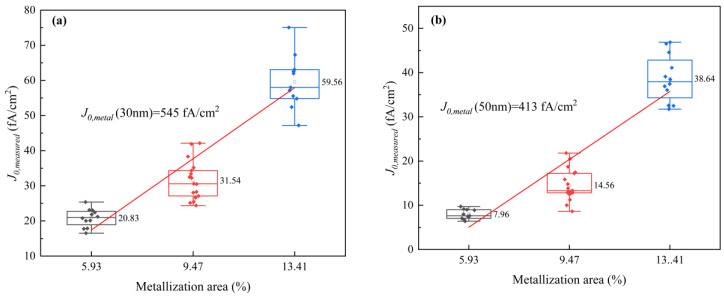

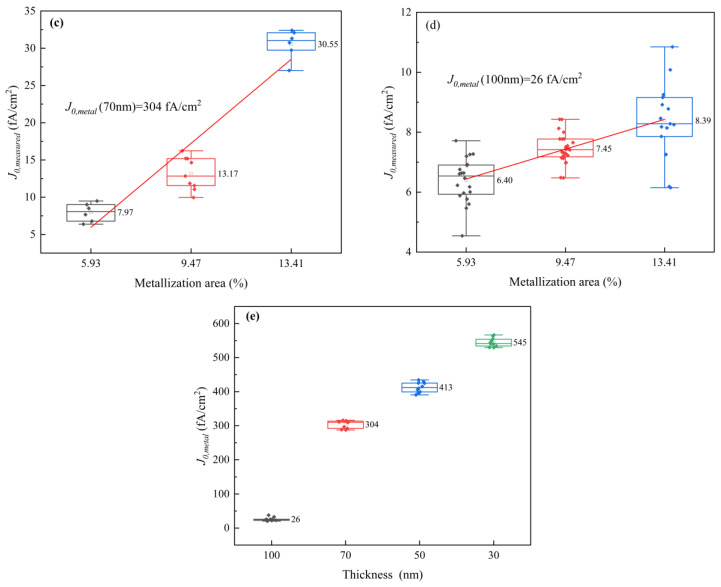
*J*_0,measured_ plots for n^+^-poly-Si layers (**a**) 30 nm, (**b**) 50 nm, (**c**) 70 nm, and (**d**) 100 nm thick with different metallization fractions ranging from 5.9% to 17.3% and (**e**) *J*_0,*metal*_ of different thickness of n^+^-poly-Si layers. (The solid pattern represents the test data, and the hollow pattern represents the average value).

*J*_0,measured_ plots of n^+^-poly-Si layers of various thicknesses with different metallization fractions are depicted in Figure 4. The values of *J*_0,*metal*_ are determined through a straightforward linear interpolation of the measured data points [42,43,44]. The results indicate that the n^+^-poly-Si layers with a thickness of 100 nm have the lowest *J*_0,measured_ values, approximately 26 fA/cm^2^. This could be attributed to the thick layer’s ability to withstand the depth of the corrosion of the poly-Si layer by Ag paste. The thinner the poly layer, the more it is destroyed by the paste, which greatly reduces the passivation performance. However, when the thickness of the layer was decreased from 70 to 30 nm, *J*_0,*metal*_ increased from 304 to 545 fA/cm^2^. It is well known that metal contact recombination is related to doping concentration and corrosion depth. When the poly-Si layer thickness is more than 70 nm, the doping concentration increases (Figure 2a), and a thick poly-Si layer provides good resistance against the corrosion of the slurry. When the poly-Si layer is thin, it cannot meet the demand for metallization. The thinner the poly layer, the more the paste destroys the poly layer, which greatly reduces the passivation performance.

**Figure 5:**
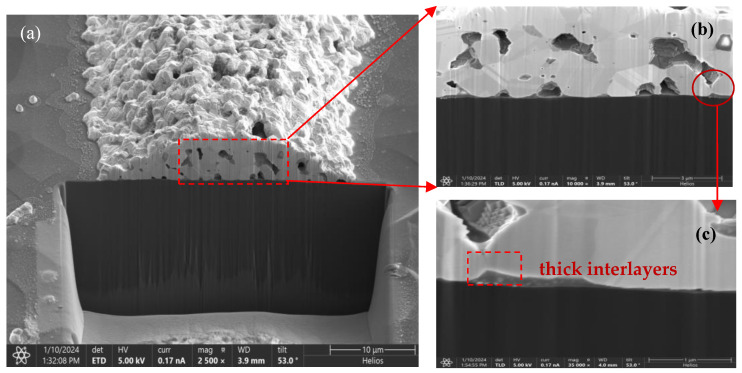
Cross-sectional SEM images of the metal contact on (**a**) an n^+^-poly-Si layer, the interface of Ag–Si fingers (**b**); and numerous little white particles on the local area of the surface of the poly-Si layer (**c**) shown in (**b**).

**Figure 6:**
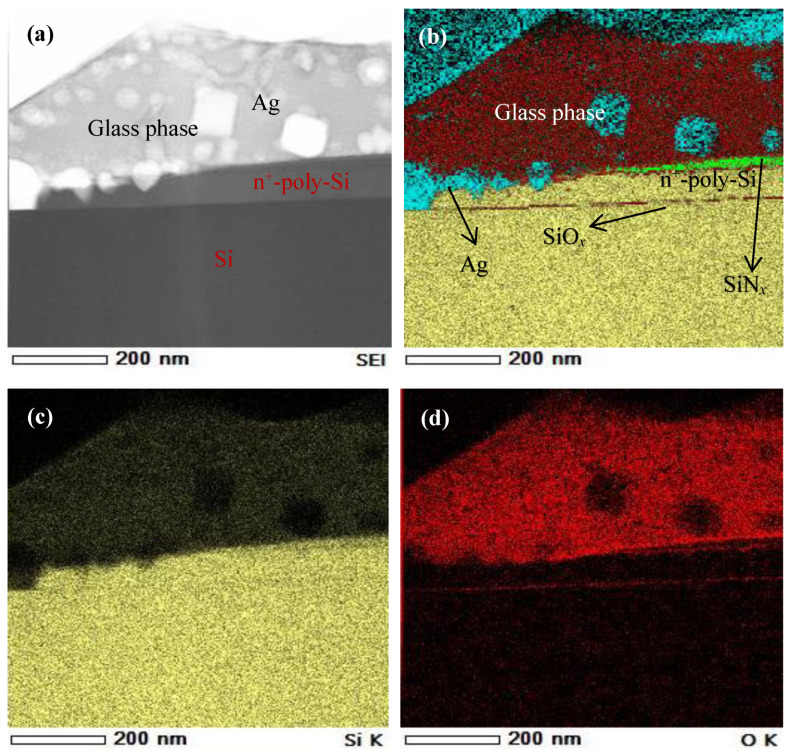

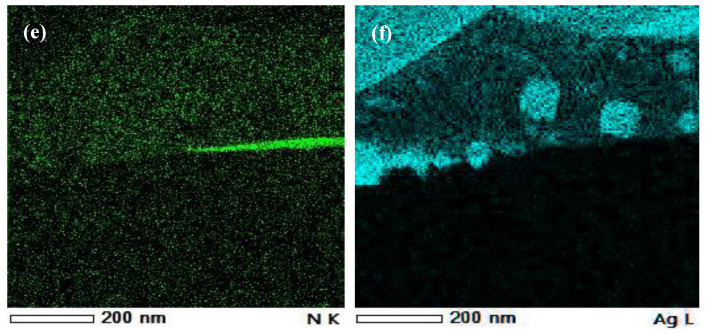
(**a**) HAADF-STEM image of the formed contact of an n+ poly-Si layer from Figure 5c; (**b**) corresponding EDX elemental mapping and EDX elemental mapping of Si (**c**), O (**d**), N (**e**), and Ag (**f**).

Cross-sectional SEM images of the interface between the screen-printed Ag bulk and the n^+^-poly-Si/SiO*_x_* contacts are shown in Figure 5. The polished cross-section of the cell shows a 100 nm thick n^+^-poly-Si layer uniformly covered by the glass layer beneath the Ag bulk, with some voids. The interface between a Ag finger and the n^+^-poly Si/SiO*_x_* layer is obvious in Figure 5a; it can be observed that the Ag bulks have a block structure and that the interlayer n^+^-poly Si/SiO*_x_* layer seems to be non-uniform (Figure 5b). The thickness of the interlayer is thin in some regions; however, there are some thick interlayers in the dotted-line region (Figure 5c). It is hypothesized that the numerous small white particles on the local area of the poly-Si layer’s surface are the result of Ag particle precipitation. Meanwhile, we further show the chemical composition of the thick interlayer in Figure 5c, the EDX elemental mapping images are displayed in Figure 6. 

A large number of white schistose Ag particles embedded in the SiO*x*-based glass phase are visible in a HAADF-STEM (High-Angle Annular Dark-Field Scanning Transmission Electron Microscopy, HITCHI, Japan) image of the contact interface between the Ag grid finger and the polished surface of the n^+^-poly-Si layer (Figure 6a). The corresponding elemental distribution was measured using EDX spectroscopy and is shown in Figure 6b as an overlay of Ag, Si, N, and O; the matrix elements are shown separately in Figure 6b–f. The partial SiN*_x_* layer can still be observed at the interface between the Ag finger and the n^+^-poly-Si/SiO*_x_* layer, which could influence the contact performance (Figure 6e). Some Ag particles have corroded the poly-Si layer, which is crucial for metallization recombination (Figure 6f). However, the SiO*_x_* tunneling layer is clearly present in Figure 6b,d. 

**Figure 7:**
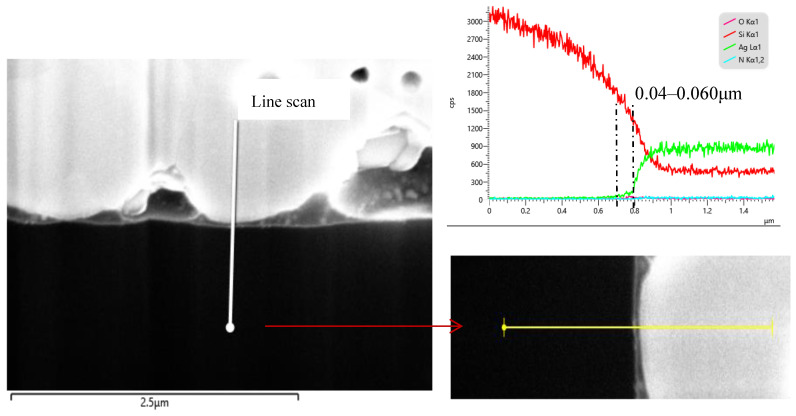
STEM/EDX energy element line scan images of the Ag–Si interface.

As shown in the EDX line scan (Figure 7), the corrosion depth of the Ag particles is approximately 0.06 μm, which is consistent with the *J*_0*.metal*_ analysis mentioned above. This imposes a constraint on the minimum thickness of the n^+^-poly-Si layer. Therefore, when the thickness of the poly-Si layer is thin, it cannot resist the corrosion caused by the Ag paste. This leads to the formation of metal–semiconductor contact to some extent, resulting in a higher *J*_0,*metal*_.

**Figure 8:**
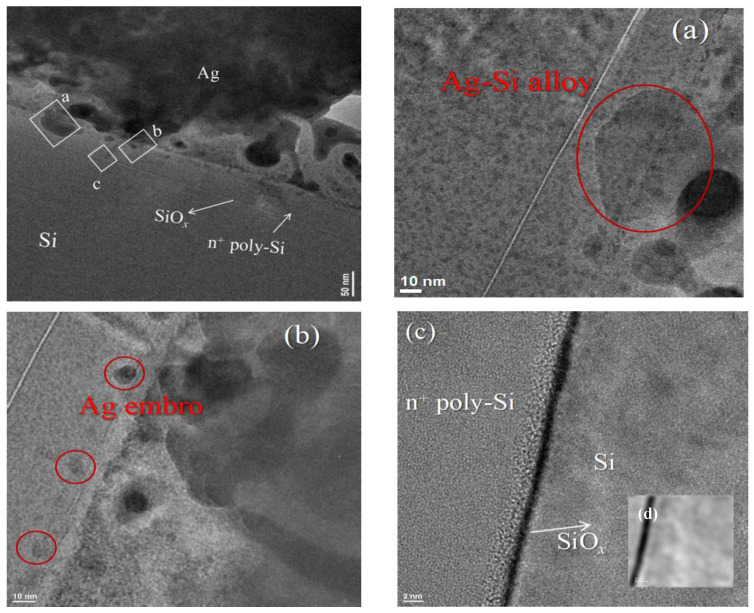
STEM bright-field cross-sectional images of Ag bulk/glass layer/50nm n^+^ poly-Si/SiO*_x_* layer contact; high-density of Ag embryos on SiN*_x_* layer was observed in (**a**). Ag–Si alloy in the n^+^ poly-Si layer (**b**), (**c**) Ag-embryo on n^+^ poly-Si layer, and (**d**) selected area of tunneling layer SiO*_x_*.

TEM was conducted and revealed the composition of the contact cross-section formed between the screen-printed Ag bulk and the n^+^-poly-Si/SiO*_x_* layers. As shown in Figure 8, the thickness of the n^+^-poly-Si/SiO*_x_* layer is 50 nm. The Ag–Si alloy can be observed in specific regions of the n^+^-poly-Si layer which are only 10 nm away from the SiO*_x_* layer. This means that the depth of the Ag–Si alloy can reach up to 40 nm, which is consistent with Figure 7. There are also some Ag embryos in certain areas of the n^+^-poly-Si layer (Figure 8b). This can explain why the contact resistance is low but the metallization recombination increased. However, the Ag embryos did not penetrate the Si substrate; instead, a clear SiO*_x_* layer can be observed (Figure 8c). The above analysis indicates that under the current process conditions, the thickness of the n^+^-poly-Si layer should be more than 40 nm.

**Figure 9:**
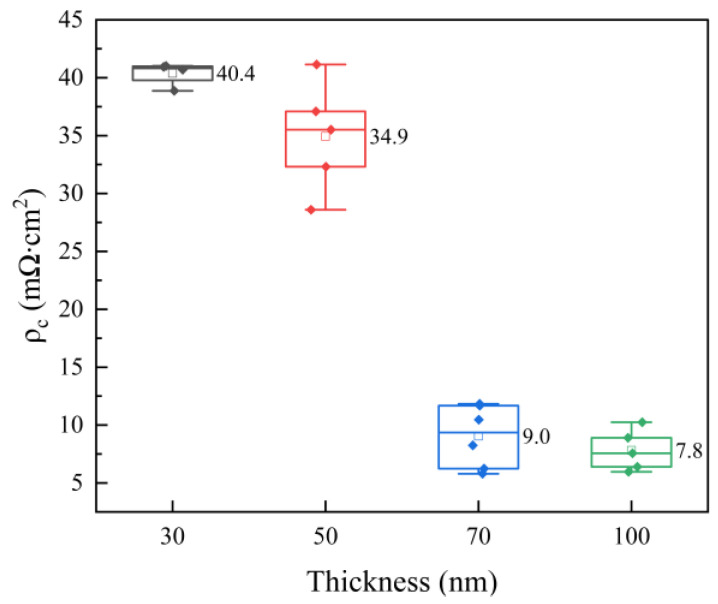
Plot of contact resistivity of solar cells.

The contact resistivity of the solar cells with varying n^+^-poly-Si layer thicknesses is depicted in Figure 9. There is a remarkable change in *ρ_c_*, which decreases from 40.4 to 7.8 mΩ·cm^2^ with an increase in the layer thickness from 30 nm to 100 nm. Due to an increase in the total doping concentration, the width of the internal depletion region in a silicon wafer can be narrowed, enabling the quantum mechanical tunneling of charge carriers through Schottky barriers [47].

### 3.2. I–V Parameters 

**Figure 10:**
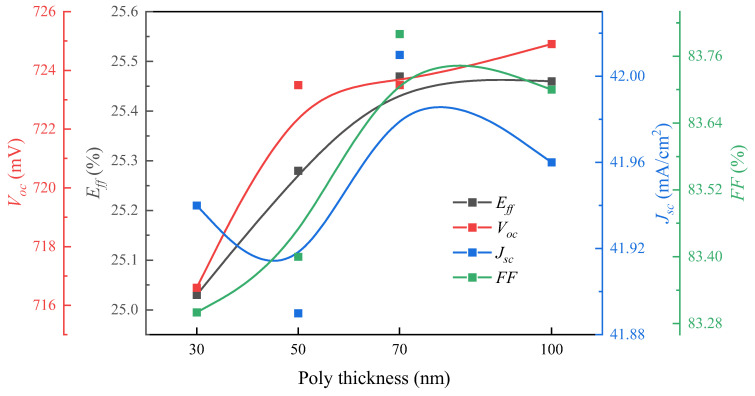
*I*–*V* parameters of n-TOPCon solar cells.

Table 2 lists the *I*–*V* parameters of the cells as a function of the thickness of the n^+^-poly-Si layer. The cells with 70 nm and 100 nm thick n^+^-poly-Si layers have the same efficiency, approximately 25.45%, which is mainly due to the current gain compensating for the loss in *V_oc_*. When the thickness of n^+^-poly-Si layer is equal to 70 nm, the cells have a high *J*_0,*metal*_ value, but the cells only exhibit a slight decrease in the *V_oc_* value of 1 mV and have the best FF. This result is consistent with the aforementioned analysis of the *ρ_c_* value. However, as shown in Figure 10, when the thickness of the n^+^-poly-Si layer ranged from 30 to 50 nm, there was a sharp decline in the efficiency of the cells by over 0.2%. There is a slight decrease in the *V_oc_* of 1 mV for the cells with 50 nm thick n^+^-poly-Si layers, while there is a significant decrease in the *V_oc_* of 8 mV for the cells with 30 nm thick n^+^-poly-Si layers. This occurred because the silver embryos penetrated the passivation layer of SiO*_x_*. Meanwhile, the *J_sc_* value of the cell does not exhibit any advantages with the decreased thickness of the poly-Si layer. The decrease in the passivation performance of the n^+^-poly-Si layer is primarily attributed to Ag corrosion, which impacts the generation of photogenerated electrons. Based on the above data, it can be inferred that the optimal thickness of the n^+^-poly-Si layer of cell for industrial production is 70 nm.

### 3.3. Failure Analysis

**Figure 11:**
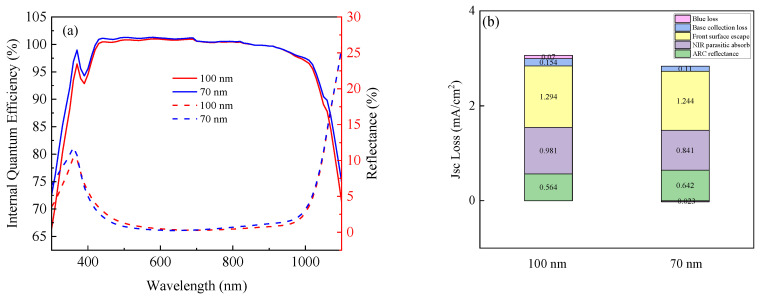
Solid line curves show internal quantum efficiency (IQE), and dashed line curves show optical refection (**a**) and current loss mechanisms of cells with 70 nm and 100 nm thick n^+^ poly-Si layers (**b**).

Current loss was investigated to understand the effects of the n^+^-poly-Si layer thickness. As shown in Figure 11a, there is no significant difference in optical reflection between the 70 nm and 100 nm samples, as represented by a dashed curve. The solid line curve represents the internal quantum efficiency (IQE). A notable disparity in the IQE between 70 nm and 100 nm is observed at short wavelengths of <550 nm. The blue responses of these sizes were notably enhanced by the low total phosphorus doping concentration. The IQE for the 70 nm cell exhibited a good response at wavelengths < 900 nm. The curve shows that the values of the *J_sc_* are increased by decreasing the thickness of the poly-Si layer owing to the low degree of parasitic absorption on the rear surface.

A current loss analysis of n-TOPCon cells with 70 nm and 100 nm thick n^+^-poly-Si layers is shown in Figure 11b. The cells with an n^+^-poly-Si layer thickness ranging from 70 nm to 100 nm have three advantages. The first is the “NIR parasitic absorption loss”, which results in an increase of 0.14 mA/cm^2^ in the *J_sc_* value due to the thin n^+^-poly-Si layer. The other advantages are “blue loss” and “base collection loss”, which benefit from the low total dopant concentration of P. However, there is one disadvantage of “ARC reflectance” for the cells with a 70 nm thick n^+^-poly-Si layer. This issue is related to the wraparound thin poly-Si layer, which is may damage the texture of the front surface and increase the reflectance [48]. 

After process optimization, the thin poly-Si layer can lead to an increase in *J_sc_* value. However, it is essential to strike a balance between maximizing the *J_sc_* value and minimizing the recombination effect of metallization on the etched depth of the poly-Si layer. Therefore, the development in metallization of industrialized poly-Si selective emitter technology will be the next focus of research. 

## 4. Conclusions

*J*_0_, *J*_0,*metal*_, *ρ_c_*, and microstructure analyses of contact formation were conducted, and the *I*–*V* parameters of solar cells were investigated as functions of the n^+^-poly-Si layer thickness. We introduced an n^+^-poly-Si layer with a thickness of 70 nm and a surface concentration of 5 × 10^20^ atoms/cm^3^ to enhance the conversion efficiency to 25.65%. The results showed that the *J*_0_ values of n^+^-poly-Si layers exhibit a minor change of approximately 0.5 fA/cm^2^ when the thickness ranges from 30 to 100 nm. However, the thickness of the n^+^-poly-Si layer had a significant impact on *J*_0,*metal*_ values, which increased from 304 to 545 fA/cm^2^, and *ρ_c_* values, which decreased from 9 to 40.4 mΩ·cm^2^, when the thickness decreased from 70 to 30 nm. The analysis above indicates that the thickness of the n^+^-poly-Si layer cannot be less than 40 nm under current process conditions. This limitation is primarily attributed to the presence of corroded silver (Ag) particles at a certain depth within the n^+^-poly-Si layer. A reduction in the thickness of the poly-Si layer by 30–50 nm did not result in an increase in the short-circuit current density, resulting in an inability to effectively collect current. The *ρ_c_* and *J*_0,*metal*_ results, along with the *I*–*V* characteristics, indicate that the thickness of the n^+^-poly-Si layer needs to exceed the depth of Ag particle corrosion to achieve a low *ρ_c_*. Simultaneously, it is crucial to ensure that the *J*_0,*metal*_ value is low. This optimized n^+^-poly-Si layer process can be commercially applied in photovoltaics to reduce the processing time and thus lower costs. 

## Figures and Tables

**Table 1:** Process conditions and performance of wafers.

*Conditions* (*Poly Thickness*)	*SiO_x_ Layer Formation Process*	*a-Si Layer Formation Process*				*Phosphorus Doped Poly-Si Layer Formation Process*				*Main Result Data*			
	*t*_oxidation_ (min)	*T* (°C)	*t* (min)	*G_SiH_*_4_ (sccm)	ECV (nm)	*T_deposition_* (°C)	*G_POCl3_* (sccm)	*T_drive-in_* (°C)	*R_□_* (Ω/sq)	*ρ_c_* (mΩ·cm^2^)	*J_0_* (fA/cm^2^)	PL (a.u)	*J*_0,*metal*_ (fA/cm^2^)
**100 nm**	8	600	24	850	100	850	1100–1200	850–860	45	2.5	4.6	31,090	26
**70 nm**			19		70				52	2.9	4.1	32,258	304
**50 nm**			16		50				54	3.5	4.7	33,262	413
**30 nm**			13		30				57	3.9	4.7	33,216	545

*t*_oxidation_: post-oxidation duration; *T*: temperature; *t*: time; *T*_drive-in_: drive-in temperature; *ρ_c_*: contact resistivity; *J*_0_: emitter dark saturation current density; *G*: gas flow.

**Table 2:** *I–V* parameters of n-TOPCon solar cells.

Poly Thickness (nm)	*E_ff_* (%)	*V_oc_* (mV)	*J_sc_* (mA/cm^2^)	*FF* (%)	Cell Area (cm^2^)
30	25.03	716.6	41.94	83.3	334.88
50	25.27	723.5	41.89	83.4	
70	25.47	723.5	42.01	83.8	
100	25.46	724.9	41.96	83.7	

*E_ff_*: efficiency; *V_oc_*: open circuit voltage; *J_sc_*: short-circuit current density; *FF*: fill factor.

## Data Availability

Data is contained within the article.
